# Prospective cohort study of the predictive value of inflammatory biomarkers over clinical variables in children and young people with cancer presenting with fever and neutropenia

**DOI:** 10.12688/f1000research.73075.1

**Published:** 2021-10-22

**Authors:** Bob Phillips

**Affiliations:** 1 Centre for Reviews and Dissemination, University of York, York, Yorkshire, YO10 5DD, UK; 2 Regional Department of Paediatric Haematology and Oncology, Leeds Teaching Hospitals NHS Trust, Leeds, LS1 9TX, UK

**Keywords:** febrile neutropenia, neutropenic sepsis, childhood cancer, inflammatory biomarkers, IL6, IL8, procalcitonin

## Abstract

Introduction

Fever during chemotherapy induced neutropenia is a common and potentially life-threatening complication of the treatment of childhood cancer. Predictions of poor outcome could be enhanced by incorporating serum biomarkers of inflammation at presentation and reassessment.

Methods

A prospective cohort study was conducted of children under 18 years old, being treated for cancer or a cancer-like condition, who presented with fever (≥ 38.0°C) and neutropenia (neutrophil count < 0.5*10 ^9^/L). Clinical features were recorded, along with three experimental inflammatory biomarkers: procalcitonin (PCT), interleukin-6 (IL-6) and interleukin-8 (IL-8). Outcomes included serious medical complications (SMC): any infection related mortality, critical care and organ support, severe sepsis, septic shock, significant microbiologically defined infection, or radiologically confirmed pneumonia.

Results

Biomarker assessments were undertaken in 43 episodes of fever and neutropenia, from 31 patients aged between four months and 17 years old (median six years): 20 were female and 22 had acute leukaemia. Five episodes of SMC were noted. PCT, IL-6 and IL-8 had poor individual discriminatory ability (C-statistic 0.48 to 0.60) and did not add to the value of clinical risk stratification tools. Insufficient data were collected to formally assess the value of repeated assessments.

Conclusions

Incorporating serum biomarkers of inflammation at presentation of episodes of fever with neutropenia in childhood does not clearly improve risk stratification. Repeated assessments over time may be of value.

## Introduction

Infection, frequently presenting as fever during chemotherapy induced neutropenia, is a common and potentially life-threatening complication of the treatment of childhood cancer. Modern management approaches have promoted the use of clinical decision rules to stratify patients at low risk of serious medical complications during febrile neutropenia (FN), enabling them to be safely managed with less intensive therapy as an outpatient. ^1^

In addition to the role of clinical risk stratification, there is an increased desire to use modern biochemical markers of inflammation in predicting the risk of severe sepsis. There is increasing interest in inflammatory biomarkers such as procalcitonin (PCT), an inflammatory marker that has been shown to rise in response to bacterial infection in non-immunosuppressed children, ^2^ and various cytokines including interleukin (IL)-6 and IL-8 to improve the diagnostic accuracy of a prediction rule in children with FN. ^3^

A systematic review and meta-analysis of the discriminatory ability of biomarkers in children with FN ^4^^,^^5^ concluded that while several small studies suggest PCT, IL-6 and IL-8 may be valuable for predicting severe infection in children with FN, the true impact remains unknown. A smaller number of studies explored the role of serial (i.e., 0 h, 12–24 h, 48 h) biomarkers to detect documented infection or sepsis. In one study, the difference between mean C-reactive protein (CRP), PCT and IL-8 at 24 hours in children with and without sepsis was more pronounced than at presentation. ^6^ These data suggest the optimal value for prediction may be made by incorporating biomarkers at early reassessment (i.e., within 12–24 h or 24–48 h), rather than at presentation.

This study aimed to undertake a focused analysis of three promising inflammatory biomarkers: PCT, IL-6 and IL-8 in both initial and value of serial testing and their additional discriminatory value above routine clinical features using the PICNICC model ^7^ and AUS-score. ^8^

## Methods

This study took place in Leeds Children’s Hospital between March 2016 and March 2018. It recruited patients on presentation of FN or pre-enrolled them during routine appointments where they, or their parents or guardians, affirmed their consent in written form. In addition to formal information sheets, a link to an animated video summary of the research was provided https://www.youtube.com/watch?v=Z1AXzJqatds. ^9^ Ethical approval was given by the Leeds West NHS Research Ethics Committee [15/YH/0357].

Children could be included who were: younger than 18 years old, who had cancer, or received a stem cell transplant, or who had Langerhans cell histiocytosis in need of cytotoxic chemotherapy, or haemophagocytic lymphohistiocytosis undergoing active treatment, or severe aplastic anaemia, and attended with febrile neutropenia. Fever was defined as temperature ≥ 38.0°C and neutropenia, an absolute neutrophil count ≤ 0.5 g/L.

All patients were admitted to hospital and managed according to institutional FN pathways; these consisted of full evaluation and empiric piperacillin/tazobactam (or suitable alternatives) until afebrile 48 hours and with no other reason for antimicrobials, regardless of neutrophil count. Inflammatory biomarkers, with the exception of CRP, were analysed in batches and their results masked from the treating team. Using this unselective approach, with treatment unaffected by the results of the biomarker analyses, we minimized the biases which can arise through cherry-picking of patients and altering medical treatment on the basis of the test under evaluation (selection and incorporation biases).

Demographic data, variables and outcomes included core items as devised by the International Paediatric Fever in Neutropenia Working Group (see Extended Data, Table 1) ^9^. Clinical assessments and blood samples for biomarkers were taken at presentation (within 12 h of fever or admission) and daily until the end of the FN episode or discharge as part of usual clinical care.

**Table 1.** Distribution of diagnosis in 31 patients with biomarker data.

Diagnosis	Number of patients
Acute leukaemias	21
Other haematological malignancies	2
Solid tumours	5
Brain tumours	3

The primary outcome measure was ‘serious medical complication (SMC)’ ^10^ (defined by any of (i) infection related mortality (ii) admission to ICU/HDU/other ward/unit for organ support (iii) severe sepsis or (iv) septic shock) or significant microbiologically defined infection, or radiologically confirmed pneumonia. Secondary outcomes measures were the component parts of the primary outcome, death within 30 days, infection related mortality, clinically defined infection, infection with antibiotic-resistant bacteria and relapse of primary infection. Initial power calculations estimated ~400 episodes would detect an additional benefit C-statistic discriminatory of +0.10.

Results were analysed descriptively, and assessment made of the individual discriminatory value of the inflammatory biomarkers using the C-statistic for SMC, and their additional value to clinical prediction rules (PICNICC prediction and AUS-score). Analyses were undertaken using base R version 3.6.0 (R: A language and environment for statistical computing. R Foundation for Statistical Computing, Vienna, Austria) (RRID:SCR_001905) and the package pROC (pROC: an open-source package for R and S+ to analyze and compare ROC curves) Further analysis of the results was planned to be by hierarchical logistic regression modelling of episodes within patients, assessing the predictive value of clinical and then biomarkers of inflammation at admission, day one and subsequent timepoints. Additional assessments of the sensitivity and specificity of the markers, alone and in combination, were also proposed.

## Results

Data was collected from 65 patients with an episode of fever and neutropenia. Of those, 43 episodes in 31 patients had biomarker samples taken, with 31 episodes with ‘day one’ data. The distribution of diagnoses is shown in Table 1. The patients ranged from four months to 17 years old (median six years), and 20 were female.

Of these 31 episodes, five were assigned an SMC: two significant viral infections with oxygen requirement, and one each of: *Fusobacterium* blood stream infection, *Escherichia coli* urinary tract infection, and culture-negative severe sepsis requiring fluid boluses. No patient received intensive care support or died. There were eight non-serious viral infections and one non-serious central line colonization.

**Figure 1.**
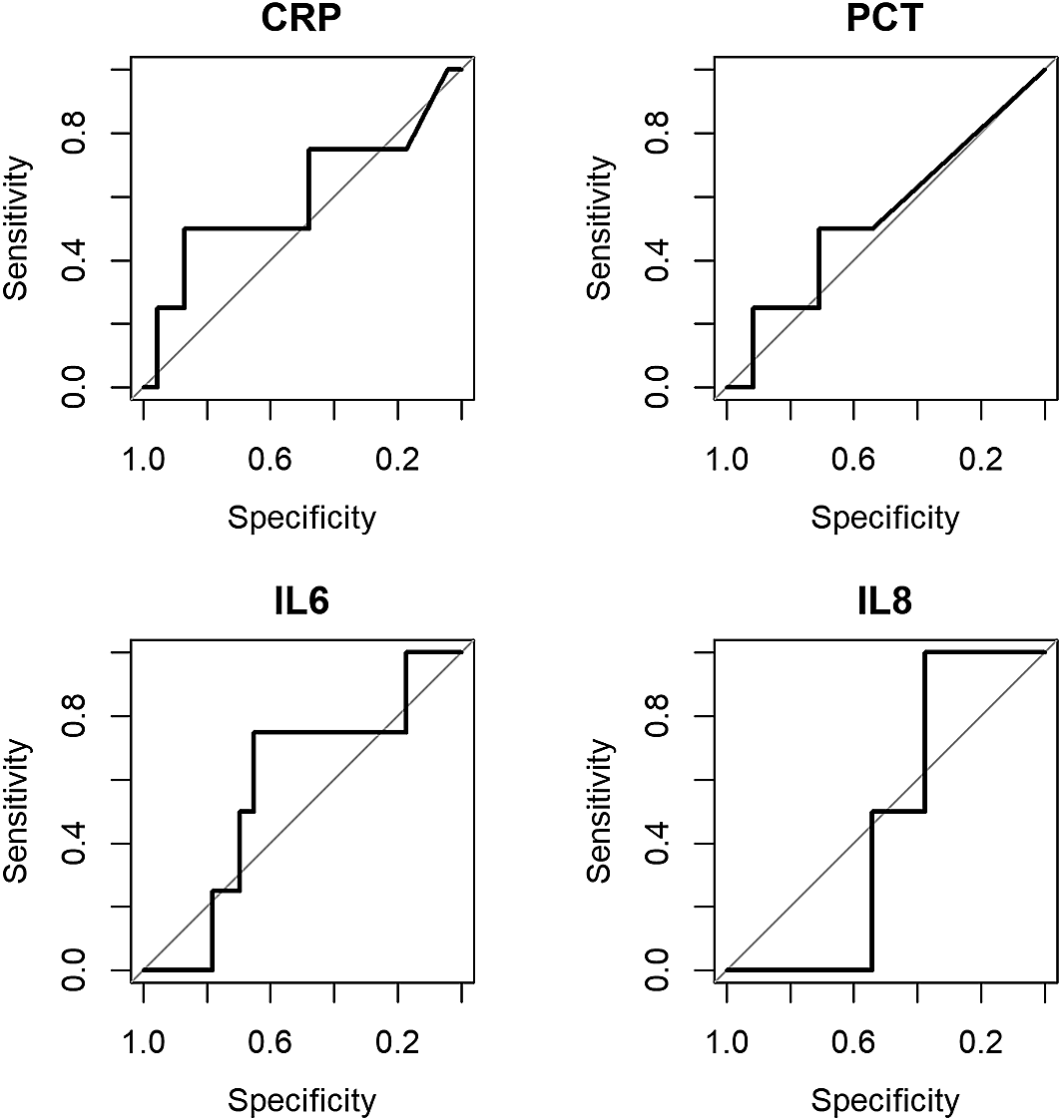
Plots of discriminatory values of admission biomarkers in distinguishing patients with serious medical complication (SMC). CRP = C-reactive protein; PCT = procalcitonin; IL-6 = interleukin-6; IL-8 = interleukin-8.

All biomarkers performed poorly in distinguishing those who developed an SMC (see Figure 1), with C-statistic estimates ranging from 0.48 to 0.60 (see Table 2). The biomarkers were ineffective in improving the discriminatory value of the PICNICC risk prediction (change in C-statistic: −0.10 to +0.04), and only marginally useful in improving the simpler AUS-score (+0.04 to +0.18) (See Extended Data, Table 2) ^9^ Given the paucity of data and lack of diagnostic value, no complex analyses were undertaken.

**Table 2.** C-statistic values for biomarkers to distinguish patients with serious medical complication (SMC). CRP = C-reactive protein; PCT = procalcitonin; IL-6 = interleukin-6; IL-8 = interleukin-8.

Biomarker		With PICNICC prediction	With AUS-score
CRP	0.60	0.69	0.71
PCT	0.54	0.55	0.59
IL-6	0.57	0.55	0.57
IL-8	0.48	0.64	0.58
*Rule alone*		0.65	0.53

The biomarker levels concurred better with the physician assessment of “severe” clinical illness, though in no case was this statistically significantly different (see Table 3, p > 0.10).

**Table 3.** Mean biomarker levels in patients who did, and did not, appear clinically unwell at presentation. CRP = C-reactive protein; PCT = procalcitonin; IL-6 = interleukin-6; IL-8 = interleukin-8.

Biomarker	Mean ‘severe’ clinical appearance (n = 5)	Mean ‘non-severe’ clinical appearance (n = 26)
CRP	111 mg/dL	42 mg/dL
PCT	5.8 ng/ml	0.03 ng/ml
IL-6	105 pg/ml	67 pg/ml
IL-8	210 pg/ml	176 pg/ml

Repeated measures of the biomarkers were available in 25 episodes. Development of SMC did not occur in patients who presented with non-severe symptoms and consistently low inflammatory biomarkers despite ongoing fevers. Of those with SMC, 4/5 had reductions in biomarker levels as their infection resolved; they stayed high in the one case of culture negative severe sepsis.

## Discussion

This prospective study of inflammatory biomarkers in paediatric febrile neutropenia found little support for their use as indicators of covert infection. Technical and administrative challenges limited the number of samples collected during the study, despite enthusiasm from the patients and their families. This adds to the body of evidence describing a relatively limited role in initial stratification.

Serial use of these markers, where they can be used to suggest an infection is under control, or controlled, is an area of active testing. PCT has been studied in several patient groups to diagnose sepsis and monitor response to treatment. A systematic review ^11^ and subsequent large (>1500 patients) RCT with pragmatic study design examining PCT-guided decision-making in neonates (NeoPIns) with suspected infection and critically ill adults on ICU (SAPS) found significant reductions in antibiotic duration in the PCT arm. ^12^^,^^13^ Two large UK studies looking at the same question in adults and children with suspected or proven serious bacterial infections are ongoing. ^14^^,^^15^ These studies specifically exclude immunocompromised patients such as children with cancer, and work exploring this is required to enhance patient experience and reduce antimicrobial resistance. ^16^^–^^18^

The results of this study should dissuade clinicians from routinely using inflammatory biomarkers in making initial stratification of children with febrile neutropenia. The onward investigation of their serial use in antimicrobial stewardship should be pursued within carefully monitored studies.

## Data availability

### Underlying data

The data for this study contains the following elements, which when combined would make the patients identifiable given the rarity of childhood cancer in the identified geographical area and time of this study:

Demographic data: (ii) episode date; (iii) age; (iv) cancer diagnosis & treatment.

FN episode data: (i) inpatient or outpatient onset; (ii) time of start of temperature; (iii) time of presentation.

A reduced dataset taking out all items to produce sufficient anonymity (for sensitive data; childhood cancer) would severely limit their utility for researchers.

The patients and families who took part in this study, and the Research Ethics Committee who granted permission for it, agreed the data should be available for sharing in ethically approved secondary use projects. Such studies are typically individual participant data meta-analysis collaboratives. Anyone who has such an approved project, investigating aspects of paediatric febrile neutropenia and biomarker profiles, is encouraged to approach the author at bob.phillips@york.ac.uk for access to the dataset.

### Extended data

Open Science Framework (OSF): Extended data for ‘Prospective cohort study of the predictive value of inflammatory biomarkers over clinical variables in children and young people with cancer presenting with fever and neutropenia’, https://www.doi.org/10.17605/OSF.IO/CVFZB. ^9^

This project contains the following extended data:

• Extended Data Tables.docx (Extended Table 1: Data items collected and Extended Table 2: AUC-ROC (C-statistic) values for biomarkers to distinguish patients with SMC)
• Consent Video (mp4 format)

Data are available under the terms of the Creative Commons Zero “No rights reserved” data waiver (CC0 1.0 Public domain dedication).

## Consent

Written informed consent for publication of the patients’ details was obtained from the patients/parents/guardian of the patient.
