# Supplementary material for: Prospective cohort study of the predictive value of inflammatory biomarkers over clinical variables in children and young people with cancer presenting with fever and neutropenia
Source: F1000Res. 2022 Feb 3;10:1070. Originally published 2021 Oct 22. [Version 2] doi: 10.12688/f1000research.73075.2 (PMC8831847; doi:10.12688/f1000research.73075.2)
Supplement: Peer review history

# Peer review: Reviewer response for version 2

**DOI:** 10.5256/f1000research.120929.r122269

**Published:** 2022-02-10

**Authors:** Paul J. Gibson

**Affiliations:** 1 Department of Pediatrics, McMaster University, Hamilton, Ontario, Canada

Thanks to the author for his work and his edits in response to the review. No further suggestions.

Is the work clearly and accurately presented and does it cite the current literature?

Yes

If applicable, is the statistical analysis and its interpretation appropriate?

I cannot comment. A qualified statistician is required.

Are all the source data underlying the results available to ensure full reproducibility?

Partly

Is the study design appropriate and is the work technically sound?

Yes

Are the conclusions drawn adequately supported by the results?

Yes

Are sufficient details of methods and analysis provided to allow replication by others?

Yes

Reviewer Expertise:

I am a clinical pediatric oncologist with research experience in supportive care. I cannot however, confidently suggest all statistical methods quoted in the paper are appropriate.

I confirm that I have read this submission and believe that I have an appropriate level of expertise to confirm that it is of an acceptable scientific standard.

# Peer review: Reviewer response for version 2

**DOI:** 10.5256/f1000research.120929.r122270

**Published:** 2022-02-07

**Authors:** Rejin Kebudi

**Affiliations:** 1 Division of Pediatric Hematology-Oncology, Oncology Institute, Istanbul University, Istanbul, Turkey

The paper is very valuable in its revised form.

 Approved.

Is the work clearly and accurately presented and does it cite the current literature?

Yes

If applicable, is the statistical analysis and its interpretation appropriate?

Yes

Are all the source data underlying the results available to ensure full reproducibility?

Yes

Is the study design appropriate and is the work technically sound?

Yes

Are the conclusions drawn adequately supported by the results?

Yes

Are sufficient details of methods and analysis provided to allow replication by others?

Yes

Reviewer Expertise:

Pediatric oncology

I confirm that I have read this submission and believe that I have an appropriate level of expertise to confirm that it is of an acceptable scientific standard.

# Peer review: Reviewer response for version 1

**DOI:** 10.5256/f1000research.76698.r100188

**Published:** 2022-01-21

**Authors:** Rejin Kebudi

**Affiliations:** 1 Division of Pediatric Hematology-Oncology, Oncology Institute, Istanbul University, Istanbul, Turkey

The manuscript *Prospective cohort study of the predictive value of inflammatory biomarkers over clinical variables in children and young people with cancer presenting with fever and neutropenia* investigates the role of PCT, IL6, IL8 at first day and serial monitoring in paediatric febrile neutropenic episodes.

 The unselective approach for the biomarkers (the inflammatory biomarker results except for CRP were masked from the treating team) minimized the biases for medical treatment. Did the results of CRP (which the medical team could see) effect treatment decisions?

 There were 43 episodes and 31 patients, only 31 episodes had first day data. Despite that, the biomarker levels concurred better with the physician assessment of severe clinical illness, however, this was not statistically significant. The conclusion was that at presentation of febrile neutropenic episodes, serum biomarkers of inflammation do not improve the risk stratification, repeated assessment over time may be of value.

 Were there any differences in patients who had acute leukemia vs solid tumors? Were all of the febrile neutropenic episodes, in which biomarkers were investigated, high risk? If there were both high risk and low risk episodes, did the biomarkers differ within the risk groups?

 In table 3 the mean inflammatory biomarker values in severe clinical appearance are numerically much higher than the non-severe ones; what was the range of each biomarker in the severe and non-severe group. Do the authors define the severe clinical appearance (table 3) the same as SMC?

 Although the use of these biomarkers were not statistically significant in initial stratification, the evaluation of a larger cohort with higher number of SMC, may give more information on the value of the initial inflammatory biomarkers for predicting a SMC or sepsis. A larger cohort may also give more information for the value of the use of serial biomarker monitoring and which one/ones to use cost-effectively. The discussion may include some of these concerns.

 Do see this for further reference. ^1^

Is the work clearly and accurately presented and does it cite the current literature?

Yes

If applicable, is the statistical analysis and its interpretation appropriate?

Yes

Are all the source data underlying the results available to ensure full reproducibility?

Yes

Is the study design appropriate and is the work technically sound?

Yes

Are the conclusions drawn adequately supported by the results?

Yes

Are sufficient details of methods and analysis provided to allow replication by others?

Yes

Reviewer Expertise:

Pediatric oncology

I confirm that I have read this submission and believe that I have an appropriate level of expertise to confirm that it is of an acceptable scientific standard.

## References

[1] : Guideline for the management of fever and neutropenia in children with cancer and/or undergoing hematopoietic stem-cell transplantation. *J Clin Oncol* .2012;30(35) : 10.1200/JCO.2012.42.7161 4427-38 10.1200/JCO.2012.42.7161 22987086

# Response

**Published:** 2022-02-01

**Authors:** Bob Phillips

**Affiliations:** Centre for Reviews and Dissemination, University of York, UK

There are a number of interesting questions raised in the review:

1. Were there any differences in patients who had acute leukemia vs solid tumors?

**This is a very fair question, and no, there were no qualitative differences in patient type. Given the small numbers, multiple analyses for variation were felt to be unwise and only the highest level (risk-grouping and biomarkers) were undertaken.**

1. Were all of the febrile neutropenic episodes, in which biomarkers were investigated, high risk? If there were both high risk and low risk episodes, did the biomarkers differ within the risk groups?

**The risk groups are shown in Extended Data Table 2; they included all levels of risk.**

1. In table 3 the mean inflammatory biomarker values in severe clinical appearance are numerically much higher than the non-severe ones; what was the range of each biomarker in the severe and non-severe group.

**This is a good question and Table 3 has been modified to add the (range) as well as the mean values.**

1.  Do the authors define the severe clinical appearance (table 3) the same as SMC?

**No – severe clinical appearance and significant medical complication are defined differently. SMC is defined in the fifth methods paragraph, “severe clinical appearance” is the gestalt impression of the examining physician.**

# Peer review: Reviewer response for version 1

**DOI:** 10.5256/f1000research.76698.r97615

**Published:** 2021-11-10

**Authors:** Paul J. Gibson

**Affiliations:** 1 Department of Pediatrics, McMaster University, Hamilton, Ontario, Canada

**General Comments:**

 This is a nice small study that tackles an interesting and still unanswered question with regards to the utility of biomarkers in the risk stratification of Pediatric Febrile Neutropenia. However, it appears there were challenges in recruitment and sample collection that are not specifically outlined. It may be helpful to describe those challenges to guide future efforts. 

**Abstract**:

 Conclusions: "Repeated assessments over time may be of value". I'm not convinced that the body of the paper nor the discussion justify this statement based on the results of this study. While other studies are referenced that may point to the utility of serial assessments, the concept is not substantially discussed in this paper.

**Introduction**:

 The second paragraph mentions interest in newer biomarkers but fails to address CRP until it used as a single example in the 3rd paragraph. I suggest the authors make earlier reference to this 'older' biomarker and its role (or lack thereof) in risk stratification and highlight why PCT IL-6 and IL-8 may be preferable. 

**Methods**:

 "Using this unselective approach, with treatment unaffected by the results of the biomarker analyses, we minimized the biases which can arise through cherry-picking of patients and altering medical treatment on the basis of the test under evaluation (selection and incorporation biases)". This sentence may be more succinctly stated. such as "PCT, IL-6 and IL-8 values were not provided to clinicians to avoid bias"

 As Table 1 states results, it is more appropriate in the results section.

 The methods reference power calculations suggesting ~400 episodes were required. but only 43 were captured, yet there is no explanation for planned duration or reason for termination of the study. 

**Results**

 Data was collected on 65 patients, yet only 31 have had biomarkers taken? Why? Did they refuse collection? Did they present outside of times feasible for biomarker collection? The Discussion references technical and administrative challenges, but maybe expand what you mean? 

 You may consider showing graphically the changes in biomarkers in the 5 SMC patients.

Is the work clearly and accurately presented and does it cite the current literature?

Yes

If applicable, is the statistical analysis and its interpretation appropriate?

I cannot comment. A qualified statistician is required.

Are all the source data underlying the results available to ensure full reproducibility?

Partly

Is the study design appropriate and is the work technically sound?

Yes

Are the conclusions drawn adequately supported by the results?

Yes

Are sufficient details of methods and analysis provided to allow replication by others?

Yes

Reviewer Expertise:

I am a clinical pediatric oncologist with research experience in supportive care. I cannot however, confidently suggest all statistical methods quoted in the paper are appropriate.

I confirm that I have read this submission and believe that I have an appropriate level of expertise to confirm that it is of an acceptable scientific standard, however I have significant reservations, as outlined above.

# Response

**Published:** 2022-02-01

**Authors:** Bob Phillips

**Affiliations:** Centre for Reviews and Dissemination, University of York, UK

"General Comments:

1. This is a nice small study that tackles an interesting and still unanswered question with regards to the utility of biomarkers in the risk stratification of Pediatric Febrile Neutropenia. However, it appears there were challenges in recruitment and sample collection that are not specifically outlined. It may be helpful to describe those challenges to guide future efforts."

**This is an extremely good point, and paragraph 3 has been added to the Discussion.**

 "Abstract:

1. Conclusions: "Repeated assessments over time may be of value". I'm not convinced that the body of the paper nor the discussion justify this statement based on the results of this study. While other studies are referenced that may point to the utility of serial assessments, the concept is not substantially discussed in this paper."

**This is also reasonable – the few data there are congruent but an overstatement from this report. Modified.**

 "Introduction:

1. The second paragraph mentions interest in newer biomarkers but fails to address CRP until it used as a single example in the 3rd paragraph. I suggest the authors make earlier reference to this 'older' biomarker and its role (or lack thereof) in risk stratification and highlight why PCT IL-6 and IL-8 may be preferable. "

**A worthwhile negative to explicitly state. Added to into paragraph 3 to explain why the newer ones are possibly better.**

 "Methods:

1. "Using this unselective approach, with treatment unaffected by the results of the biomarker analyses, we minimized the biases which can arise through cherry-picking of patients and altering medical treatment on the basis of the test under evaluation (selection and incorporation biases)". This sentence may be more succinctly stated. such as "PCT, IL-6 and IL-8 values were not provided to clinicians to avoid bias"

**This is definitely true, but I’d really like to keep the longer explanation. As a tutor of critical appraisal, I have found the word “bias” is often thrown about as a correct answer, but more knowing it’s the code to use than the meaning of it. Additionally, it conforms more strongly to the STROBE checkpoint 9.**

1. As Table 1 states results, it is more appropriate in the results section.

**Fair. This is now a supplementary Table, and Table 1 is within the results.**

 The methods reference power calculations suggesting ~400 episodes were required. but only 43 were captured, yet there is no explanation for planned duration or reason for termination of the study. 

**Again, reasonable request for the missing information. Added in Methods and Discussion.**

 "Results:

1. Data was collected on 65 patients, yet only 31 have had biomarkers taken? Why? Did they refuse collection? Did they present outside of times feasible for biomarker collection? The Discussion references technical and administrative challenges, but maybe expand what you mean? "

**Agreed – discussion paragraph added.**

 Final Comment:

- You may consider showing graphically the changes in biomarkers in the 5 SMC patients.

**It is definitely worth considering. Given the very small numbers and the lack of real value of the work in serial measurements arising from this report, as correctly emphasised earlier, adding a graph might well over-egg this part of the pudding again and is probably better left text-based.**
